# Recapture Lysosomal Enzyme Deficiency via Targeted Gene Disruption in the Human Near-Haploid Cell Line HAP1

**DOI:** 10.3390/genes12071076

**Published:** 2021-07-15

**Authors:** Annie Brown, Jiayi Zhang, Brendan Lawler, Biao Lu

**Affiliations:** Department of Bioengineering, School of Engineering, Santa Clara University, 500 El Camino Real, Santa Clara, CA 95053, USA; akbrown@alumni.scu.edu (A.B.); jzhang10@scu.edu (J.Z.); blawler@scu.edu (B.L.)

**Keywords:** β-glucocerebrosidase, Gaucher disease, lysosomal storage disorder, disease model, CRISPR-Cas9

## Abstract

Background: Advancement in genome engineering enables rapid and targeted disruption of any coding sequences to study gene functions or establish human disease models. We explored whether this approach can be used to study Gaucher disease, one of the most common types of lysosomal storage diseases (LSDs) in a near-haploid human cell line (HAP1). Results: CRISPR-Cas9 targeting to coding sequences of β-glucocerebrosidase (GBA), the causative gene of Gaucher disease, resulted in an insertional mutation and premature termination of GBA. We confirmed the GBA knockout at both the gene and enzyme levels by genotyping and GBA enzymatic assay. Characterization of the knockout line showed no significant changes in cell morphology and growth. Lysosomal staining revealed more granular lysosomes in the cytosol of the GBA-knockout line compared to its parental control. Flow cytometry analysis further confirmed that more lysosomes accumulated in the cytosol of the knockout line, recapturing the disease phenotype. Finally, we showed that this knockout cell line could be used to evaluate a replacement therapy by recombinant human GBA. Conclusions: Targeted gene disruption in human HAP1 cells enables rapid establishment of the Gaucher model to capture the key pathology and to test replacement therapy. We expect that this streamlined method can be used to generate human disease models of other LSDs, most of which are still lacking both appropriate human disease models and specific treatments to date.

## 1. Introduction

Gaucher disease is one of the most common types of lysosomal storage diseases (LSDs), which comprise over 50 hereditary metabolic disorders [1,2,3]. LSDs occur due to genetic mutations that result in the deficiency or reduced activity of native lysosomal enzymes [4,5]. These enzyme defects lead to the accumulation of specific macromolecules in various cells, causing tissue damage and organ failure [5]. Although enzyme replacement therapy (ERT) is now available for six LSDs, including Gaucher disease, it is prohibitively expensive and only effective in mild to moderate patients, leaving those with severe neurological complications untreated [6,7]. To address these unmet medical needs, streamlined methods for the generation of robust human disease models are needed to speed up disease research and drug discovery [6]. To this end, we intend to develop a robust method to rapidly establish human LSD cell models that could be more efficient than existing animal or patient-derived models, which have significant limitations. For example, although a number of LSD mouse models exist, they are expensive to maintain and may not replicate human diseases faithfully due to species-specific differences [8,9,10]. Diseased cells isolated from patients may provide clinically relevant cell models; however, their sources are limited and their cultures may be difficult to maintain with restricted passages. In addition, their weaker phenotypes due to varied mutations and lack of parental controls make them less useful for disease study and drug development. We argue that the establishment of more robust LSD models using targeted genome editing tools in established cultured human cells could offer an important solution to these problems. Specific disruption of LSD associated genes using new genetic tools could result in a drastic decrease in enzyme activities in these immortal cell lines, yielding more robust cell models with perfect parental controls.

Recent advancement in genome editing technologies based on CRISPR-Cas9 has enabled targeted disruption of single genes in living human cells to study cellular functions and model diseases [11,12,13,14]. Instead of studying gene function when taken out of the context of the genome, researchers can now directly ablate gene function in their endogenous context in virtually any type of human cell, enabling them to elucidate function and pathophysiology, as well as develop new therapies. However, gene knockout in human cells is hampered by their diploid nature; there is a low efficiency in simultaneously disrupting both alleles [13]. Therefore, gene knockout using targeted nucleases, including CRISPR-Cas9, zinc finger nuclease (ZFN) or transcription activator-like effector nuclease (TALEN) requires complicated design strategies and labor-intensive screening [15,16]. To overcome these shortcomings, Carette JE et al. demonstrated an effective strategy by using haploid rather than diploid human cells to systemically interrogate gene functions [17,18]. We think that this strategy may provide a solution to generate genetic models to study human diseases such as LSDs.

Gaucher disease is an important and well-characterized autosomal recessive disorder caused by mutations in the acid β-glucocerebrosidase (GBA) gene [19,20]. The hallmark of this disease is defective GBA with an associated increase in lysosomes; however, introduction of functional GBA can effectively reverse the pathology [21,22]. In this feasibility study, we describe a streamlined method in establishing a Gaucher disease model by using a CRISPR-Cas9 based strategy in a human haploid cell line (HAP1). We showed a targeted disruption of human GBA in an engineered HAP1 line. Importantly, the targeted disruption resulted in a near total loss of GBA enzymatic activity, capitulating the hallmark of Gaucher disease and its associated lysosomal abnormality. Finally, we demonstrated that recombinant human GBA can effectively reverse enzyme deficiency in this disease model. Because lysosomal enzymes share similar domain structures, our study opens up a new path to rapidly generate additional LSD models. The robustness of this new system could speed up the development of new therapies for the most difficult-to-treat metabolic disorders.

## 2. Materials and Methods

### 2.1. Materials

Chemicals including sodium citrate dihydrate, citric acid, sodium cholate and DTT were purchased from Sigma Aldrich (St. Louis, MO, USA). LysoTracker Red DND-99 and IMDM medium were purchased from Thermo Fisher Scientific (Waltham, MA, USA). Custom primers were synthesized by IDT (Coralville, IA, USA). Recombinant human GBA was purchased from R&D Systems (Minneapolis, MN, USA). Reporter 5xlysis buffer was purchased from Promega (Madison, WI, USA). InstaGene Matrix for genomic DNA extraction and PCR was purchased from Bio-Rad Laboratories (Hercules, CA, USA). Fetal bovine serum was purchased from GE Healthcare (Chicago, IL, USA).

### 2.2. Cell Culture

CRISPR-Cas9 modified GBA-knockout and its parental HAP1 cells were obtained from Horizon Discovery (Cambridge, UK). These cells were maintained in IMDM supplemented with 10% fetal bovine serum, 2 mM of GlutaMax and 100 U/mL of penicillin-streptomycin. Typically, cells were maintained at ~40–90% confluency and passaged every 2–3 days at a recommended ratio of 1 to 10.

### 2.3. GBA Knockout Strategy

The human GBA gene is composed of 11 exons and encodes a lysosomal enzyme of 536 amino-acids. As the aim was to knockout GBA enzyme activity, the conservative enzymatic domain was chosen. The guide RNA targeting the coding sequences in Exon 6 likely generated indels of GBA loci. By transfection with CRISPR-Cas9 into HAP1, cells were cloned and a frameshift line was screened out by PCR amplification and followed by double-stranded DNA sequencing. It is important to note that gRNA targeting Exon 6 may also potentially alter its pseudogene GBAP1. However, this specific complication may not occur when targeting other LSD enzymes without corresponding pseudogenes. Successful GBA targeting and enzymatic assay were used to further validate a functional knockout of GBA gene via CRISPR-Cas9.

### 2.4. Genomic DNA Extraction and PCR

Genomic DNAs were extracted from both parental and GBA-knockout lines using InstaGene Matrix reagent (Bio-Rad Laboratories). To confirm GBA gene disruption, the targeted gene loci were PCR amplified using the primer pair: forward- CTGATGGAGTGGGCAAGAT; reverse- AAGTGATAAGCAGAGTCCCATACTC. PCR products with sizes of 425 bp (wildtype) and 1232 bp (insertion) were expected in the case of targeted gene disruption by non-homologous end joining. PCR reactions were performed using 2xTaq Master Mix (GenScript; Piscataway, NJ, USA) with a hot start at 95 °C for 2 min, followed by 35 cycles of denature at 95 °C for 30 s, annealing at 60 °C for 20 s and extension at 72 °C for 1 min. Products were electrophoresed in 1% agarose gel to confirm the size of reactions.

### 2.5. GBA Enzyme Assay

GBA activity was determined fluorometrically using 4-methy-lumbelliferyl-beta-D-glucopyranodide (NBD-Glu) as a substrate, as previously reported [23]. For each assay, the NBD-Glu solution was freshly prepared, and positive (rhGBA) and negative (bovine albumin) controls were included. Briefly, whole cell lysates were prepared from cultured HPA1 cells by adding 100 µL of reporter lysis buffer (Promega) to each well. Lifted cells were subject to freeze–thaw cycles at −80 °C. Typically, the enzyme reaction was carried out in a volume of 50 µL, using 25 µL whole cell lysate in a pH 6 solution containing 50 mM sodium citrate, 25 mM sodium cholate and 5 mM DTT. The reaction plate was incubated for 30 min at 37 °C and stopped by adding 50 µL of stop solution (0.5 M glycine, 0.3 M NaOH, pH 10). The fluorescent signal at a wavelength of 365 nm excitation and 445 nm emission was recorded using a TECAN infinite M200PRO plate reader.

### 2.6. Lysosome Staining

Cells were fluorescently stained by a lysosome staining solution of LysoTracker Red DND 99. The cell culture medium was replaced with a staining solution (50 nM LysoTracker Red DND 99) for 30 min at 37 °C. The staining solution was replaced with a PBS buffer and imaged immediately or analyzed via flow cytometry.

### 2.7. Fluorescent and Confocal Microscopy

Live cells were monitored and recorded using an Olympus fluorescence microscope (Waltham, MA, USA) or Leica TCS SP8 confocal microscope. To show the intracellular lysosomes, we recorded both fluorescent and transmitted light images from the same field. Any image modifications, including brightness and contrast, were applied to the entire image using instrument software. Cells stained with the fluorescent lysosome staining solution were analyzed using an Accuri C6 Plus Flow Cytometer (BD Biosciences, San Jose, CA, USA), as reported. In brief, cells grown at 80–90% confluence were stained and washed 3 times with PBS. Over 10,000 events for each sample were recorded to quantify the levels of lysosome using a red fluorescence channel.

### 2.8. Data Collection and Statistical Analysis

Student’s two-tailed *t*-test was used to determine the statistical significance of our studies, with *p*-values < 0.05 being considered significant. We expressed the values for all measurements as mean ± standard deviation.

## 3. Results

### 3.1. System Design and Knockout Strategy to Model Human Gaucher Disease

**Figure 1:**
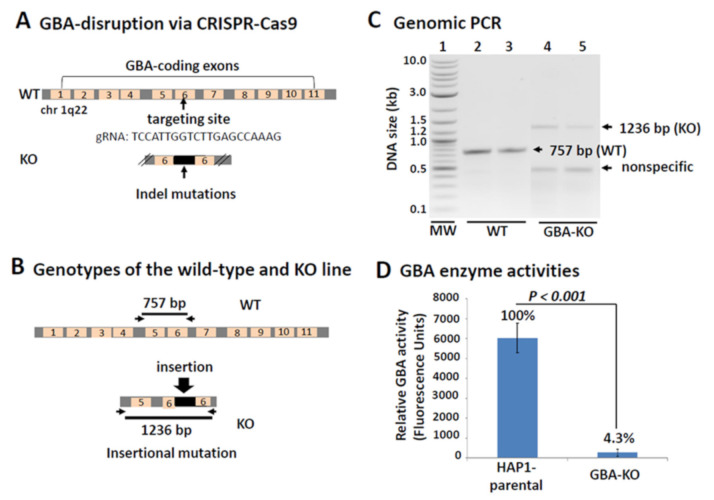
Schematic overview of the targeting strategy to knockout GBA in the human genome. (**A**) The human GBA gene is composed of 11 exons located on chromosome 1. The guide RNA (gRNA) of CRISPR-Cas9 was designed to target the coding region in Exon 6. Introns are shown in gray; exons are in yellow and are numbered. The arrow indicates the binding site of gRNA. (**B**) The locations of the PCR primers used for genotyping the HAP1 cells are indicated by arrows. This primer pair flanks the gRNA binding sites and will produce a 757 bp DNA in wild-type HAP1 and a 1236 bp DNA fragment in KO HAP1 due to a 479 bp insertion. (**C**) Genotyping analysis of the HAP1 parental and KO lines. Exon 6-specific primers amplified expected PCR products of 757 bp in the wild-type HAP1 parental line (lane 1–2) and 1236 bp in the KO HAP1 line. (**D**) A drastic reduction of GBA activity in the KO line as compared to those of wild-type HAP1.

Our goal was to test the feasibility of using CRISPR-Cas9 for the quick establishment of LSD models in the near-haploid cell line, HAP1, by disrupting the disease-associated gene. Although other genome editing tools, such as TALEN and ZFN, can be used for this purpose, Cas9 was chosen because of its simplicity, efficiency, specificity and availability [16]. In this study, we focused on the disruption of the human GBA gene. As shown in Figure 1, the human GBA gene is composed of 11 exons and encodes a lysosomal enzyme of 536 amino-acids. Similar to other lysosomal enzymes, GBA proteins start with a short signal peptide (SP, 1–30 aa), followed by a long and conservative enzyme domain (Glyco_hydro_30, 93-510 aa) [24,25]. According to its domain structure, any guide RNA targeting the coding sequences of the conservative domain will most likely generate indel mutations at the cleavage sites due to error prone repair mechanisms [16]. To ensure a complete ablation of the enzyme, it may be advantageous to target the early portion of the enzyme domain. In most cases, these indel mutations may well result in a frame-shift or premature termination at a high frequency (~66%) [16,26]. After clonal expansion, a desired clone bearing a frameshift mutation was selected and validated by PCR amplification and double-stranded DNA sequencing. For this particular clone, a guide RNA targeting Exon 6 introduced mutations to the GBA gene, resulting in a 479 bp insertion at the targeted site, which was sequence-confirmed by Horizon Discovery (Figure S1. The insertion was predicted to cause a premature termination of the GBA with a loss of 314 aa of its C-terminus (Figure S1). Conserved domain analysis revealed a significant truncation of the conserved catalytic domain, supporting a drastic decrease in its enzymatic activity [24].

To confirm the successful GBA-KO in the HAP1 knockout line, we conducted both genotyping and enzyme activity assays in the GBA-KO and parental HAP1 lines. A polymerase chain reaction using exon 6 specific primers (primer pares flanking the exon 5 and 6) yielded a single 757 bp band in the wild-type control, and an expected 1236 bp mutational band in the GBA-KO line (Figure 1C). This was consistent with a 479 bp insertion caused by the action of CRISPR-Cas9 at the targeted site. To ensure this insertional mutation would cause a decrease in GBA enzyme activity, we then carried out a GBA enzyme assay to examine if a correspondent loss of enzyme activity had occurred. As shown in Figure 1D, the relative enzyme activity was drastically decreased by more than 95% in GBA-KO cells over the controls, indicating a total loss of GBA activity. In summary, the use of CRISPR-Cas9 in a haploid human cell line can produce gene-specific mutation in knockout cells that recapitulates pathologic hallmarks of Gaucher disease: the loss of GBA enzyme activity.

### 3.2. Characterization of GBA-KO Line

**Figure 2:**
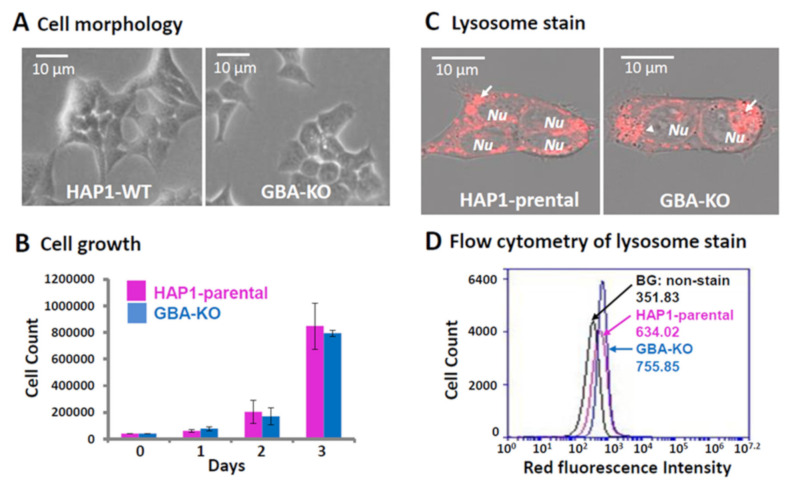
Loss of GBA and the associated changes in the GBA-KO line. (**A**) Live cell images were recorded on the cultured HAP1 and GBA-KO lines. (**B**) The same numbers of cells of either wild-type HAP1 or GBA-KO line were seeded in a 6-well plate (Day 0). The cells were cultured in a complete medium and counted for 3 consecutive days. The cell counts were mean ± standard deviation (*n* = 3). (**C**) Cells that were revived and cultured for at least two passages were stained and imaged by a confocal microscope in the wild-type and GBA-KO lines.** The lysosome staining images are overlays of fluorescent and transmitted light images using a confocal microscope (magnification of 400×). Lysosomes were red granules in the cytosolic compartment of both cultured HAP1 lines. (**D**) Flow cytometry analysis showed that the red fluorescent intensity was elevated to 755.85 in the GBA-KO line, as compared to 634.02 in the wild-type HAP1. The background red florescence was 351.83 in the unstained HAP1 cells.

CRISPR-Cas9 induced mutation of GBA results in a significant loss of enzyme activity and recapitulates defects of GBA enzymes in Gaucher disease. A loss of GBA activity results in the accumulation of glucosylceramide and glucosylphingosine in the lysosome, leading to lysosomal dysfunction [4]. However, such phenotypical changes may be highly variable in different types of cells [4]. For example, metabolically active cells, such as macrophages and osteoblasts, are most affected. Other cell types may not present any significant changes, although lower GBA activity and lysosomal accumulation may be detected. To examine whether any changes may occur in our GBA-KO lines, we first conducted a cell morphology and growth study. HAP1 is a near-haploid human cell line that was derived from the male chronic myelogenous leukemia cell line KBM-7 [17,18]. After reviving and culturing for two passages, HAP1 cells are adherent with fibroblast-like morphology. Consistent with its haploid nature, we observed a smaller cell size and nucleus (Figure 2A). We did not observe any significant changes in cell morphology and growth behavior in the GBA-KO line as compared to the HAP1 parental control. To determine if GBA-KO may cause any growth changes, we monitored cell growth for 3 consecutive days by counting cell numbers. As shown in Figure 2B, we did not observe any significant differences in growth rate between GBA-KO and the parental controls.

To investigate the pathogenesis of lysosomes, we used the LysoTracker probes for labeling and tracking acidic lysosomes in living cells. The HAP1 parental cells displayed characteristic cytosolic granules, indicating normal subcellular localization of lysosomes (left panel in Figure 2C). In contrast, the GBA-KO line showed a similar pattern of granules but in a more condensed manner (right panel in Figure 2C), suggesting an accumulation of undigested lipids or a compensation in lysosome numbers. To confirm this observation, we carried out a flow cytometry analysis using the LysoTracker labelled samples. As shown in Figure 2D, specific red fluorescence signals were evident in HAP1 parental cells vs. the non-stain cells (background noise). Consistent with the findings of fluorescent microscopy, the flow cytometry analysis showed that the GBA-KO cells had a 1.4-fold increase in lysosome numbers as compared to the parental controls. These results show that the GBA activity in the KO cells was significantly decreased, leading to either an accumulation of undigested lipids, an increased number of lysosomes or both. Additionally, the lack of GBA activity in HAP1 was not sufficient to alter cell morphology or growth. Future analysis of substrate build-up and pathogenic steps in this KO line may help to shed light on the metabolic mechanisms underlying Gaucher disease.

### 3.3. Enzyme Replacement of GBA in GBA-KO Line by Recombinant Human GBA

**Figure 3:**
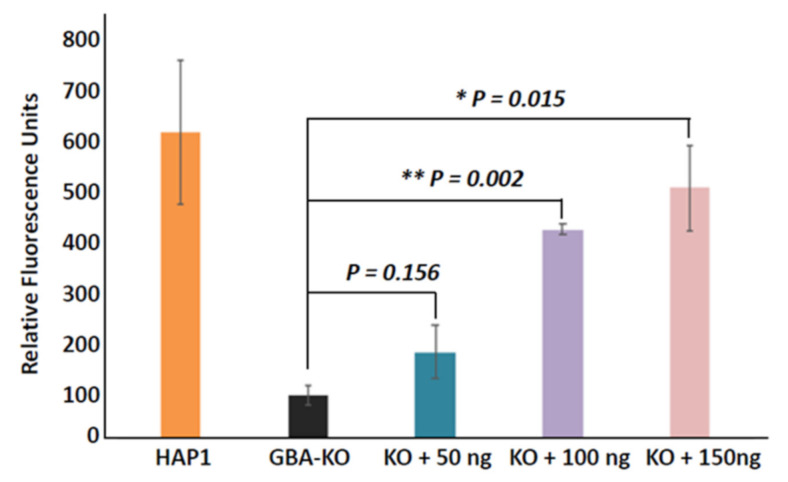
Effects of rhGBA replacement on the restoration of GBA activity in the GBA-KO cell model. The bar graph shows a significant decrease in GBA activity in the GBA-KO cells following rhGBA incubation as compared to the wild-type HAP1 cell. Treatment of GBA-KO cells with rhGBA for 24 h resulted in a dose-dependent restoration of GBA activity in GBA-KO lines. Data are mean ± standard deviation (*n* = 3). * Denotes *p* < 0.05, while ** denotes *p* < 0.01, using Student’s *t*-test.

We then investigated whether the GBA-KO line could be potentially used for drug screening. We added incremental amounts (0, 50, 100, and 150 ng) of recombinant human GBA to the culture medium of GBA-KO cells (Figure 3). After 24 h of incubation, the treatment medium was removed and the cells were washed extensively before they were lysed to prepare whole cell extraction for GBA enzyme assay. In parallel, we also included HAP1 parental cells as a positive control to represent normal levels of GBA. We found that the addition of recombinant GBA into the culture medium resulted in a dose-dependent increase in the levels of GBA in GBA-KO groups as compared to those of non-treated GBA-KO cells. At a dose of 50 ng, we observed elevated levels of GBA (~25% of HAP1 parental), although this increase did not reach statistical significance as compared to those of non-treated KO controls (Figure 3). At higher doses, significant increases in GBA activity occurred, resulting in 45% and 68% in the 100 and 150 ng groups, respectively (Figure 3). This experiment successfully mimicked the in vivo replacement therapy, indicating the potential for the use of GBA-KO cell lines in new drug screening or for comparing the efficacy of different therapeutic reagents.

## 4. Discussion

**Figure 4:**
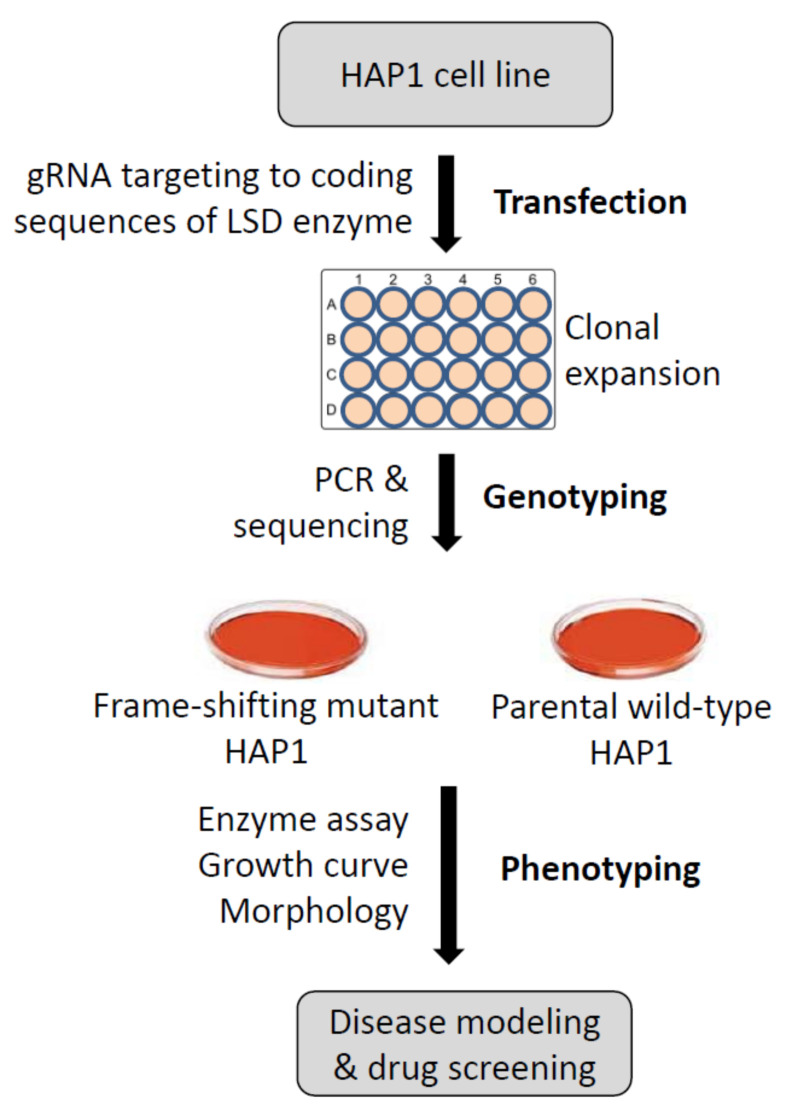
Streamlined protocols for generating LSD cell models using HAP1 cell line. Targeted gene editing using CRISPR-Cas9 enables a high rate of frame-shifting mutations and a premature termination of LSD enzymes in the near-haploid human cell line HAP1. Following initial gene editing and clonal expansion, desired enzyme mutants are obtained and confirmed by both genome typing and enzyme assays. Together with the parental HAP1, these mutants are useful resources for disease modeling and drug development of LSDs.

The combination of a near-haploid cell line with CRISPR-Cas9 genome engineering enables precise targeting of lysosomal enzymes, which proves to be an effective and streamlined method to quickly establish human hereditary disease models (Figure 4). Coupled with a parental control line, this system is useful for both studying the pathogenesis of human diseases and high throughput screening of potential therapeutics. We expect that this new approach can be easily adapted to generate additional LSD models.

To our knowledge, this is the first report that the use of HAP1 and CRISPR-Cas9 technology can achieve a significant decrease in lysosomal GBA activities. This result has very important implications for the study of human LSDs. First, HAP1 parental cells are of human origin and are more appropriate for studying metabolic changes in human genetic diseases because species-specific differences may occur in pathogenesis and symptom manifestation. Second, use of the near-haploid cell line will greatly simplify the screening procedures and at the same time effectively increase the rate of gene knockout. Third, our genotyping result and enzyme assay clearly show the total loss of GBA activities, therefore this type of disease line will be particularly useful for drug screening. Considering that only 6 out of 50 LSDs have specific treatment options, established additional disease models will speed up drug development for this important class of hereditary diseases.

We chose an enzyme assay instead of Western blotting analysis to assess any loss of GBA activities based on the following considerations. First, an enzyme assay is quantitative and may provide a more accurate assessment of the biological impact of gene mutations. Second, most LSD enzymes are heavily glycosylated and would exhibit different band patterns in various cell types or sub-clones, making the interpretation more difficult. Third, the enzyme assays we established using fluorigenic substrates are applicable to other LSD enzymes and are amenable to high-throughput screening. In contrast, Western blot analysis usually requires highly specific antibodies, which may not be available, especially for the understudied LSD enzymes. Nonetheless, it is important to note that a positive Western blot result may render additional evidence for a successful gene knockout.

In this study, we used a well-established method to determine GBA activity using fluorogenic substrates and demonstrated a significant decrease in the levels of GBA activities in the KO cells as compared to the control cells (Figure 2B). However, there are certain levels of residual activities/readings (4.3% in KO line, Figure 2B). The reasons for these residual activities/readings are not clear, but may be attributed to any of three scenarios: (1) the background noise from the fluorogenic substrates; (2) cross-reactions from other enzymes rather than GBA; and (3) residual enzyme activities from truncated GBA.

Although we have shown a significant decrease in GBA activities in the KO line, it remains to be determined whether such a decrease in GBA activities leads to a concurrent increase in the levels of toxic substrates such as glucosylceramide, glucosphingolipids and psychosine in these cells. We speculate that an accumulation of these pathogenic lipids in parenchymal cells like HAP1 may not be prominent if it occurs because substrate build-up usually occurs in the late stages of the disease, and occurs often in metabolically active macrophages but very little in parenchymal cells. Therefore, advanced analytical techniques, such as liquid chromatography-tandem mass spectrometry, are commonly used, but can be cost-prohibitive. Nevertheless, we have demonstrated that this knock-out cell line is useful to evaluate enzyme replacement therapy. Future studies on lipidomics may potentially extend the use of these engineered lines for additional applications.

Additionally, our research also points to an important direction in studying gene functions and disease pathologies in a human-relevant cell line. By using targeted nucleases, such as CRISPRE-Cas9, TALEN or ZFN, one may produce knockout lines in a similar manner as the GBA gene. This may reduce the workload as compared to those of random insertional methods by decreasing of laborious screening time and effort. Therefore, we expect that a combination of HAP1 and modern genome editing methods will provide an important fast track for basic research and translational medicine.

## Data Availability

Additional sequence data are provided in Supplementary Materials.

## Supplementary Materials

The following are available online at https://www.mdpi.com/article/10.3390/genes12071076/s1, Figure S1. (A) Conserved domain analysis of WT and KO. Insertion in Exon 6 causes a premature termination of hGBA, resulting a truncation of the conserved catalytic domain of GBA. The deduced coding sequences for both WT-GBA and GBA-KO were used to search the conserved domain database [24]. The conserved catalytic domain (glycol_hydro_30) is preserved in the WT (upper panels), but is mostly truncated in the GBA-KO line (lower panels). SP indicates the signal peptides (yellow box). The SP is predicted by SignalP-5.0 Server [25]. (B) Sequences of the 479 bp insertion after codon 222. The in-frame sequences of the insertion were underlined, which code CGNR before the stop codon of TGA.

## Abbreviations

GBA: β-glucocerebrosidase; LSD, lysosomal storage disorder; HAP1, human near-haploid cell line; CRISPR-Cas9, clustered regularly interspaced short palindromic repeats-Cas9 enzyme; PCR, Polymerase chain reaction; KO, knockout.
