# Supplementary material for: Recapture Lysosomal Enzyme Deficiency via Targeted Gene Disruption in the Human Near-Haploid Cell Line HAP1
Source: Genes (Basel). 2021 Jul 15;12(7):1076. doi: 10.3390/genes12071076 (PMC8308024; doi:10.3390/genes12071076)
Supplement: Supplementary file 1 [file genes-12-01076-s001.zip › genes-1267968-supplementary.pdf]

A Conserved domain analysis of GBA in WT and KO line

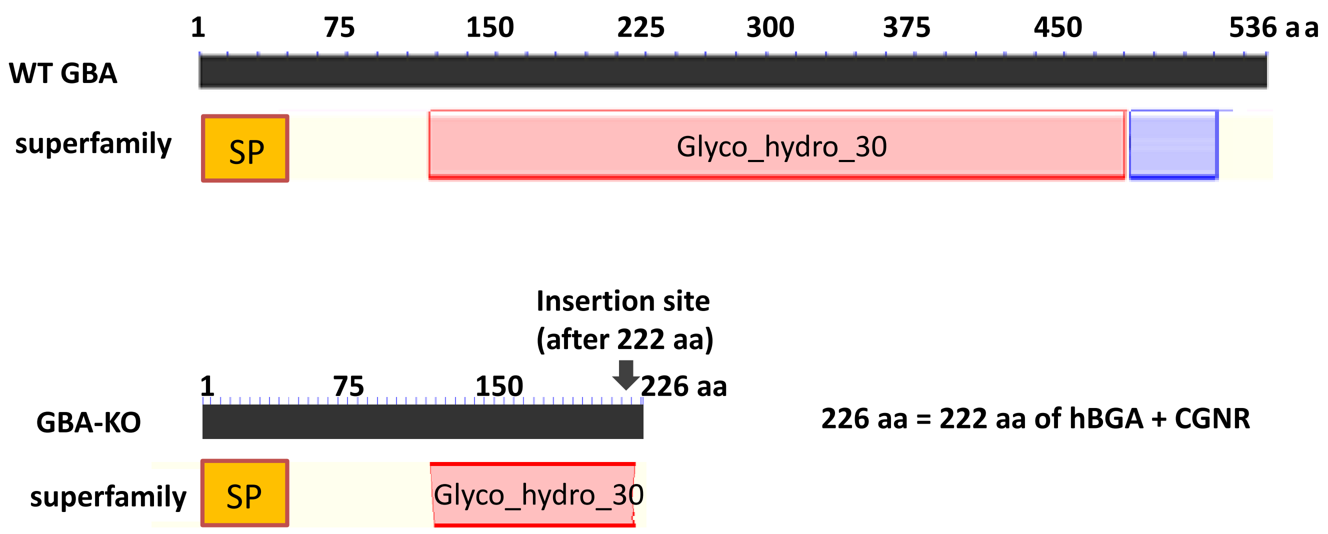

B Sequences of the 479 bp insertion

TGTGGCAACCGCTGAATATCGCCACCACCAGCGTGCTGCTGACGCTGGCCGATAACGACACGCCGGTGTGGCTTTCTACCC
CATTAAATAACGATATCGTCAACCAGAGCCTGCGTTTTCATACCAACGCGCCGCTGGTCAGCCAGCCGGAACAGGCGACCT
TCGCGGTGACGGATGAGGCGATTTCCAGCGAACAGCTCAACGCCCTTTCCACCGGCACCGCCGTTGCGCCGGAAGCGGG
TGCGACGCTGATTTTACAGGTCGCCAGCCTGAGCGGCGGACGCATGTTGCGCCTTACTGGTGCGGGTATTGCCGAAGAAC
GAATGATCGCTCCGCAGCTGCCGAAGTGCATTCTGCACGAACTCACCGAGCGCCCGCATCCGTTCCCGCTCGGCATCGACC
TGATCCTGACCTGTGGCGAGCGCCTGCTGGCTATTCCGCGAACCACTCATGTGGAGGTGTGCTGATGTACGTTGCCG

Supplementary Figure S1. (**A**) Conserved domain analysis of WT and KO. Insertion in Exon 6 causes a premature termination of hGBA, resulting a truncation of the conserved catalytic domain of GBA. The deduced coding sequences for both WT-GBA and GBA-KO were used to search the conserved domain database (Marchler-Bauer A et al. CDD/SPARCLE: functional classification of proteins via subfamily domain architectures. *Nucleic Acids Res*. 2017. 45(D)200-3). The conserved catalytic domain (glycol_hydro_30) is preserved in the WT (upper panels), but is mostly truncated in the GBA-KO line (lower panels). SP indicates the signal peptides (yellow box). The SP is predicted by SignalP-5.0 Server (Nielsen, H *et al.* A Brief History of Protein Sorting Prediction. *Protein J.* 2019. 38, 200–216). (**B**) Sequences of the 479 bp insertion after codon 222. The in-frame sequences of the insertion were underlined, which code CGNR before the stop codon of TGA.

Supplementary Figure S1
